# Clostridium difficile 027 infection in Central Italy

**DOI:** 10.1186/1471-2334-12-370

**Published:** 2012-12-22

**Authors:** Stefano Di Bella, Maria Grazia Paglia, Emma Johnson, Nicola Petrosillo

**Affiliations:** 1 National Institute for Infectious Diseases “L. Spallanzani”, Via Portuense 292, Rome, 00149, Italy; 2 Medical Microbiology Registrar, Department of Microbiology, Northern General Hospital, Sheffield Teaching Hospitals NHS Foundation Trust, Sheffield, South Yorkshire, UK

**Keywords:** *Clostridium difficile*, CDAD, NAP1, 027, Italy

## Abstract

**Background:**

*Clostridium difficile* (CD) has increasingly become recognised as a significant international health burden, often associated with the healthcare environment. The upsurge in incidence of CD coincided with the emergence of a hypervirulent strain of CD characterized as 027.

In 2010, 8 cases of CD 027 infections were identified in Italy. Since then, no further reports have been published. We describe 10 new cases of CD 027 infection occurring in Italy.

**Methods:**

Since December 2010, stool samples of patients with severe diarrhea and clinical suspicion of the presence of a hypervirulent strain, were tested for CD 027 by the Xpert *C. difficile* PCR assay (Cepheid, Sunnyvale, CA). Clinical, epidemiological and laboratory data were collected.

**Results:**

From December 2010 to April 2012, 24 faecal samples from 19 patients who fit the above criteria were submitted to our laboratory. Samples were collected from 7 different hospitals.

Of these, 17 had a positive PCR for CD and 10 were the epidemic 027 strain (59%). All PCR positive samples had a positive EIA toxin A/B test. Nine of 10 patients were recently exposed to antimicrobials and were healthcare-associated, including 4 with a history of long term care facility (LTCF) admission; the remaining case was community-associated, namely the wife of a patient with hospital-acquired CD 027 infection. Five patients experienced at least one recurrence of CD associated diarrhea (CDAD) with a total of 12 relapsing episodes. Of these, two patients had 5 and 6 relapses respectively.

We compared the 10 patients with 027 CDAD versus the 7 patients with non-027 CDAD. None of the 7 patients with non-027 CDAD had a recent history of LTCF admission and no subsequent relapses were observed (p = 0.04).

**Conclusions:**

Our study shows that CD 027 is emerging in healthcare facilities in Italy. Whilst nosocomial acquisition accounted for the majority of such cases, 4 patients had history of a recent stay in a LTCF. We highlight the substantial risks of this highly transmissible organism in such environments. Moreover, 50% of our patients with CDAD from the 027 strain had high relapse rates which may serve to further establish this strain within the Italian health and social care systems.

## Background

*Clostridium difficile* (CD) is a major cause of antibiotic-associated diarrhea (ADD) and whilst it is responsible for 15-25% of all cases of ADD, there is a greater association when severe features of disease are accounted for [1]. It predominantly affects elderly and frail hospital and nursing home patients [2] causing a broad spectrum of clinical symptoms ranging from mild diarrhea to severe life-threatening colonic perforation and toxic megacolon [3].

Over the last two decades many countries in North America and Europe have begun to register important epidemiological changes regarding CD infections and the related severity [4]. For example, Canada reported an increase in *Clostridium difficile* associated disease (CDAD) from 35.6 cases per 100,000 persons in 1991 to 156.3 per 100,000 in 2003 [5] and in the United Kingdom (UK) a six fold increase in *Clostridium difficile* infection (CDI) related mortality was observed from 1999 to 2006 [6].

This changing epidemiology in developed countries coincided with the emergence of a hypervirulent strain of CD characterized as toxinotype III, North American pulsed-field type 1, restriction-endonuclease analysis group type BI and polymerase chain reaction (PCR) ribotype 027 [7,8]. These “epidemic” strains isolated in North America and Europe appear to be genetically similar [9] and in recent months, cases of CDI caused by PCR ribotype 027 have been reported in Asia [10], providing further evidence of worldwide spread.

Associations between strain type and disease severity have been hypothesised and it is documented that when compared to other circulating strains, CD 027 is associated with a more severe disease course and a higher mortality rate [11,12]. A prospective study of CDIs conducted in Europe, spanning 14 countries, reported that patients infected with the 027 strain were three times more likely to have severe disease compared to those infected with non-027 strains [13].

In an effort to understand this strain specific virulence, bacterial factors have been evaluated during outbreaks of CDI caused by the virulent 027 strain. Increased production of toxins A and B, fluoroquinolone resistance and production of binary toxin have all been observed with this epidemic strain [2]. Indeed, this ‘hypervirulent strain’ produces up to 16 times more toxin A and 23 times more toxin B compared to non-027 circulating strains (toxinotype 0) [14].

In North America, the 027 strain accounts for 63% of health care-associated CDI [15]. Across Europe, 014/020 PCR ribotype is the commonest, with CD 027 accounting for 19 out of 389 (5%) of toxigenic isolates collected across 34 countries [16]. Of interest, none of the 027 strains were identified in Italy which is consistent with surveillance data from an earlier study; of the 50 fluoroquinolone-resistant CD clinical isolates analysed from Italian hospitals from 1985 to 2008 were analysed, none were identified as PCR ribotype 027 [17].

However, in 2010, CD 027 was identified in our country and the first documented case occurred in Northern Italy [18]. A single hospital reported 8 cases of severe CD (6% of the 130 tested CD isolates) caused by 027. Since then, no further reports have been published. In this article, we describe ten further cases of CD 027 infection occurring in central Italy over the two year period since this first documentation.

## Methods

The National Institute for Infectious Diseases in Italy, “Lazzaro Spallanzani”, serves as a national reference centre for several infectious diseases including nosocomial infections.

During the last 6 years almost 1,500 stool samples were submitted to our laboratory to be tested for CD toxins by enzyme immunoassay (EIA), with a total of 48 CDAD episodes. EIA testing for toxin A and B detection was performed by using the TechLab Tox A/B Quik Chek (rapid antigen capture; TechLab). Glutamate dehydrogenase (GDH) test is not routinely performed.

In December 2010, we procured the Xpert *C. difficile* PCR assay (Cepheid, Sunnyvale, CA). Surrounding local hospitals have the capacity to perform CD toxin testing by EIA but they refer to our centre to perform CD PCR on stool samples of patients with severe diarrhea i.e. in those who they have a clinical suspicion of the presence of a hypervirulent strain as suggested by symptom severity.

The Xpert multiplex real-time PCR assay detects toxin B gene (*tcdB*), the binary toxin gene (*cdt*), and the *tcdC* gene deletion at nucleotide 117 which is specific to the epidemic strain NAP1/BI/027. The test differentiates between the epidemic NAP1/BI/027 CD strains and other toxigenic CD strains with excellent correlation to PCR-ribotyping for detection of 027 strains [19]. The self-contained, single-use cartridge contains all the reagents necessary for the detection of *C. difficile* gene targets. Stool samples that were positive for *tcdB*, *cdt* and a single nucleotide deletion at base 117 in *tcdC* were reported as NAP1/BI/027. Samples that were positive only for *tcdB* or for *tcdB* plus *cdt* were reported as NAP1/BI/027 negative, toxigenic *C. difficile* positive .

Clinical definitions described by the European Society of Clinical Microbiology and Infectious Diseases (ESCMID) [20], by the Society for Healthcare Epidemiology of America (SHEA) and the Infectious Diseases Society of America (IDSA) [21] were used for CDAD.

CDAD was defined as diarrhea and a stool sample positive for CD toxin A and/or B, as determined using a laboratory assay. CDAD was defined as severe in patients with leukocytosis i.e. white blood cells equal to or in excess of 15,000 cells/μL and/or a serum creatinine level greater than or equal to 1.5 times the premorbid level. A complicated course of CDAD was defined as admission to an intensive care unit (ICU), surgical intervention or death associated with CDAD.

Health care-associated CDAD was defined as the development of CDAD more than 48 hours after admission or within 4 weeks after discharge. Community associated CDAD (CA-CDAD) was defined as a positive CD toxin assay in a patient with diarrhea and no documented prior overnight hospitalization or long term care facility stay in the preceeding 3 months. CDAD cases that occurred between 4 and 12 weeks after hospital discharge were defined indeterminate. The specimen should have been collected as an outpatient or within 48 hours of hospital admission. Recurrence was defined as an episode of diarrhea that occurred ≤ 8 weeks after the onset of a previous episode.

Clinical, epidemiological and laboratory data of patients with CDAD caused by CD 027 were collected. The study involved the analysis of existing clinical and laboratory data that were anonymised before being included in the study database. Thus, according to local and national regulations, this analysis was exempt from formal approval by the Ethics Committee.

Statistical analysis was performed using the *χ*2 test or the Fisher’s exact test when appropriate, and the results were considered to be significant at p < 0.05.

## Results

From December 2010 to April 2012, 24 faecal samples from 19 patients with severe diarrhea and clinical suspicion of a hypervirulent strain were submitted to our laboratory, from 7 hospitals.

Of these 19 patients, 17 had a positive PCR for CD and of these, 10 were the epidemic 027 strain (59%). All samples with a positive PCR for CD had a positive EIA test for toxin A and B.

Among the CD 027 patients, 4 were admitted in surgical units, whereas in the non-027 group only 2 were admitted in surgical units at the time of the diagnosis; the remaining patients, but one (the CA-CDAD case) were admitted in medical units.

Among the 10 CD 027 positive patients, 8 were female. The median age was 77 years (range 29–89 years). 4/10 had a history of admission to a long term care facility in the 3 months prior to the diagnosis of CDAD and overall, 9/10 were defined as health care-associated. The majority of CD 027 patients (9/10) were exposed to antimicrobials (including third generation cephalosporins and quinolones) in the 3 months preceeding diagnosis of CDI. Five out of 10 patients were taking proton pump inhibitors (PPIs). Table 1 shows the characteristics of these 10 patients, in whom the most prevalent co-morbidity was cardiovascular system disease, possibly reflecting the age distribution of these patients.

**Table 1:** Characteristics of patients with severe diarrhea tested with PCR for CD.

	**#/total patients (%)**
**Characteristic**	**CD 027**
**Gender**	
**- Female**	8/10 (80%)
**Comorbidities**	
**- Diabetes**	3/10 (30%)
**- Cardiovascular system disease**	7/10 (70%)
**- Respiratory system disease**	2/10 (20%)
**- HIV infection**	0/10 (0%)
**Inflammatory bowel disease**	1/10 (10%)
**Immunosuppressive treatment**	3/10 (30%)
**Acid-suppressant drugs**	5/10 (50%)
**Antibiotics in the prior 3 months**	9/10 (90%)
**Occult faecal blood**	5/6 (83%)
**Leukocytosis (≥ 11.000 WBCs)**	5/9 (56%)
**Hypoalbuminemia**	5/6 (83%)
**Hypogammaglobulinemia**	4/5 (80%)
**Previous LTCF stay**	4/10 (40%)
**Healthcare-associated CDAD**	9/10 (90%)
**Severe or complicated CDAD**	6/10 (60%)
**Recurrence of CDAD**	5/10 (50%)

The total of patients was referred to those whose data were available.

*CDAD*: *Clostridium difficile*-associated disease; *LTCF*: long term care facility; *WBCs*: white blood cells.

Among the 027 infected group, one patient had chronic hepatitis C. Among the non-027 group there were: 1 patient with pulmonary tuberculosis, 1 AIDS patient with tuberculous meningitis and urinary tract infection, and 1 AIDS patient with extrapulmonary tuberculosis (lymph nodes) and esophageal candidiasis. No patients were diagnosed as having any bacterial, protozoan or elmintic enteric infection.

Five out of 10, three of whom were taking PPIs, experienced at least one recurrence of CDI with a total of 12 relapsing episodes. Of these, two patients had 4 and 5 relapses respectively. Amongst the five patients who experienced recurrence episodes, 4 were female.

The management of the 10 patients with *C. difficile* 027 infection was as follows: 2 patients were treated with oral metronidazole alone; one patient was treated with oral vancomycin alone; 7 patients were treated with metronidazole and vancomycin in combination (# = 4) or sequentially (# = 3). No patients died. Both patients treated only with metronidazole had one recurrence each. The other 10 recurrences occurred in the patients treated with both vancomycin and metronidazole

The single community associated-CD patient was the wife of a patient who had hospital acquired CD 027 infection. She had no exposure to antimicrobials in the previous 3 months and developed diarrhea 5 days after her husband was discharged from hospital. All other usual aetiologies of acute diarrhoea were excluded by conventional cultures and parasitological examinations.

One patient had a concurrent diagnosis of ulcerative colitis and represents the first report of a patient with inflammatory bowel disease (IBD) with CD 027 detected in this country. This was a female patient, with no history of previous long-term healthcare stay. At 29 years of age, this was the youngest patient in our case series.

Serum albumin measurement was available for 6/10 patients and showed that 5/6 were hypoalbuminaemic at the time of CDAD diagnosis, with a mean albumin level of 2.5 g/dl (laboratory range 3.5-5 g/dl). IgG levels were measured in 5 patients; 4 of them had low serum IgG levels, i.e. 681, 612, 714 and 910 mg/dl (laboratory range 1200–1500 mg/dl).

Five patients fulfilled the criteria for severe CDAD. A further patient was diagnosed as having complicated CDAD requiring admission to the intensive care unit. There were no patient deaths in our small cohort.

We compared the 10 patients with CDAD caused by 027 versus the 7 patients with CDAD by non-027 toxigenic strains. None of the 7 patients with severe diarrhea from CD non-027 had a history of admission in a long term care facility in the 3 months prior to the diagnosis of CDAD (p = 0.08) and no subsequent relapses of CDAD were observed (p = 0.04).

## Discussion

Our study shows that CD 027 is emerging in healthcare facilities in our region, causing a spectrum of disease severity and relapsing episodes. According to our data, 50% of patients with CDAD from CD 027 experienced at least one recurrence episode of CDAD. CDAD recurs after treatment in 8-50% of cases [22] with recent reports of an upsurge in recurrence rates in some countries, likely due to several interplaying factors [23].

Unlike in other parts of Europe, there is currently no national CD surveillance program in Italy. The first cases of CD 027 were reported in 2010 [18] and due to variations in application of clinical definitions, methodology of detection and incomplete strain specific data, we do not yet know the exact magnitude of the problem. It is likely that the small number of cases described to date represents only the ‘tip of the iceberg’ and we hypothesise that the incidence of CD 027 in our hospitals is rising in real terms. This would be consistent with the pattern of spread of this epidemic strain in other parts of the world.

Long term care facilities such as nursing homes are being increasingly recognised as important reservoirs of pathogens including multidrug resistant bacteria and CD 027, within the community. Our study reported that 40% of patients had a previous stay in a long term care facility and it is likely that the high spore load associated with these settings is contributing to the persistence of CD outside of the hospital environment.

In Italy, community onset diarrhea is rarely investigated for CD. The patient with diarrhea secondary to CD 027 in the community had a strong contact history which prompted investigation. However, we must be vigilant that, as these 10 patients with CDAD 027 were not knowingly linked in time and place i.e. direct transmission events are unlikely, the community may also act as an important reservoir for CD 027 transmission [24].

The importance of CDI amongst patients with IBD is increasingly being recognized and recent studies have demonstrated increasing rates of infection, morbidity and mortality, especially in patients with ulcerative colitis [25-27]. IBD guidelines now promote testing for CD in IBD patients experiencing a relapse of symptoms [28]. In our case series, only one patient had a concurrent diagnosis of ulcerative colitis and indeed had a super-added CD infection.

Establishing an IgG response to CD has been shown to protect against infection and prevent recurrence. Of the 4 patients with a low serum gammaglobulin level, 2 had a high burden of recurrent disease with a total of 9 relapsing episodes and another had complicated CDAD requiring ICU admission. This supports the role of humoral immunity in protecting from recurrence episodes of CDAD [2].

It has been previously documented that male patients mount an augmented serum antibody response to CDI when compared to females. Furthermore, male patients are documented to have less recurrent episodes which is consistent with our findings that, of the five patients who experienced recurrence episodes, 4 were female [29,30]. Surveillance data that provides more information about patient specific factors that may influence outcome of CDAD, may allow us to tailor future management of this infection.

Despite a small case series, we have noted important differences between our patients with CDAD from 027 versus CDAD from other toxigenic strains. Indeed, previous long term care facility admission and recurrent episodes were more common in the CD 027 group. This supports recent literature which highlights long term care facilities as an important ecological niche for CD 027 [5,31,32]. In addition, our data agrees with reports by Pepin *et al.* who document an increase in the likelihood of recurrence from 20% to 47% after infection with CD 027 [23]. Similarly, Goorhuis *et al.* found clear trends in recurrence among patients with CDAD from ribotype 027 as did a recent paper from Petrella *et al.*[33,34].

Rapid PCR diagnostics can provide accurate, real-time results regarding CD positivity and 027 strain presence. Early knowledge of an epidemic strain within our healthcare facilities, may optimise clinical management of CDAD and allow rapid enhancement of infection control practices. Halting the success of this strain in becoming the single predominant strain circulating in healthcare institutions may require such an approach.

Whilst no single test is 100% sensitive and specific for the detection of *C. difficile,* cell cytotoxin and cytotoxigenic culture reference tests are the current gold standards. A limitation of our study is that neither are available in our laboratory for confirmation of the PCR results and subsequent ribotyping was also not possible.

Our current practice is to only test stool from patients with severe diarrhea for CD 027 by the Xpert real-time PCR assay so there is the possibility that we may overestimate the true frequency of CD 027 infection among patients with CDAD in our country. However, this report represents an epidemiological alert since in a recent European survey, no CD 027 positive assays were found among Italian CDAD [16].

## Conclusions

This data confirms the emergence of CD 027 in our country and underlines the association of this strain with the increased severity of CDAD and the higher rates of recurrence episodes. Surveillance data are urgently needed in order to better assess the diffusion of CD 027 in Italy and to establish risk factors associated with the transmission of CD 027 in our healthcare facilities. Without this knowledge, we have a limited armamentarium against the emerging threat of this fastidious pathogen.

## Competing interests

None for DBS, PMG, JE. PN received honoraria as speaker from: Pfizer, Wyeth, Sanofi Aventis, Astellas, MSD, Gilead, Novartis, GSK, Johnson & Johnson, Jansen Cilag, and as member of scientifical board from MSD, Pfizer and Carefusion.

## Authors’ contributions

DBS, PN participated in the design of the study. DBS and TA participated in the acquisition of data. PN and JE participated in its coordination and helped to draft the manuscript. PMG performed molecular testing of bacterial strains. All authors read and approved the final manuscript.

## Pre-publication history

The pre-publication history for this paper can be accessed here:

http://www.biomedcentral.com/1471-2334/12/370/prepub
